# Maternal vitamin D status in pregnancy and molar incisor hypomineralisation and hypomineralised second primary molars in the offspring at 7–9 years of age: a longitudinal study

**DOI:** 10.1007/s40368-022-00712-y

**Published:** 2022-05-12

**Authors:** T. Børsting, A. Schuller, P. van Dommelen, S. N. Stafne, M. S. Skeie, A. B. Skaare, S. Mørkved, K. Å. Salvesen, A. K. Stunes, M. P. Mosti, M. K. Gustafsson, U. Syversen, T. N. Fagerhaug

**Affiliations:** 1 Center for Oral Health Services and Research, Mid-Norway (TkMidt), Trondheim, Norway; 2 Department of Public Health and Nursing, Norwegian University of Science and Technology (NTNU), Trondheim, Norway; 3 Department of Child Health, The Netherlands Organization for Applied Scientific Research (TNO), Leiden, The Netherlands; 4 Centre of Dentistry and Oral Hygiene, University Medical Center Groningen, University of Groningen, Groningen, The Netherlands; 5 Department of Clinical Dentistry, University of Bergen, Bergen, Norway; 6 Department of Paediatric Dentistry and Behavioural Science, Faculty of Dentistry, University of Oslo, Oslo, Norway; 7 Department of Clinical and Molecular Medicine, Norwegian University of Science and Technology (NTNU), Trondheim, Norway; 8 Department of Endocrinology, Trondheim University Hospital (St. Olavs Hospital), Trondheim, Norway; 9 Regional Education Center (RegUt), Helse Midt-Norge, Trondheim, Norway; 10 Department of Clinical Service, Trondheim University Hospital (St. Olavs Hospital), Trondheim, Norway; 11 Department of Obstetrics and Gynaecology, Trondheim University Hospital (St Olavs Hospital), Trondheim, Norway; 12 Medical Clinic, Trondheim University Hospital (St. Olavs Hospital), Trondheim, Norway

**Keywords:** Vitamin D, Dental caries, Enamel hypomineralisation, MIH, HSPM, Pregnancy

## Abstract

**Purpose:**

The study aimed to investigate associations between maternal vitamin D status during pregnancy and molar incisor hypomineralisation (MIH) and hypomineralised second primary molars (HSPM) among children.

**Methods:**

The study had a longitudinal design using prospectively collected data from 176 mother and child pairs. Mothers were initially recruited in a randomised controlled trial to assess a pregnancy exercise programme. Along with the 7-year follow-up, we invited the children to a dental examination. The exposure variable was maternal serum 25-hydroxyvitamin D in gestational weeks 18–22 and 32–36, categorised as insufficient (< 50 nmol/l) and sufficient (≥ 50 nmol/l). Negative binomial hurdle models were used to analyse potential associations between the exposure variables and MIH or HSPM. The models were adjusted for potential confounders.

**Results:**

Among the children (7–9 years old), 32% and 22% had at least one tooth with MIH or HSPM, respectively. A significant association was found between insufficient maternal vitamin D measured in gestational weeks 18–22 and the number of affected teeth among those with MIH at 7–9 years (adjusted RR = 1.82, 95% CI 1.13–2.93).

**Conclusion:**

Considering any limitations of the present study, it has been shown that insufficient maternal serum vitamin D at mid-pregnancy was associated with a higher number of affected teeth among the offspring with MIH at 7–9 years of age. Further prospective studies are needed to investigate whether this finding is replicable and to clarify the role of maternal vitamin D status during pregnancy and MIH, as well as HSPM, in children.

**Supplementary Information:**

The online version contains supplementary material available at 10.1007/s40368-022-00712-y.

## Introduction

Children worldwide are commonly affected by dental health problems (Petersen 2003), and in recent years molar incisor hypomineralisation (MIH) and hypomineralised second primary molars (HSPM) have gained increasing attention. Classified as developmental defects of enamel, MIH and HSPM can clinically be seen as creamy white to yellow brown hypomineralisation defects on affected teeth, which in severe cases can lead to post-eruptive enamel breakdown (PEB) (Weerheijm et al. 2015). The definition of MIH is enamel hypomineralisation of systemic origin in 1–4 of the first permanent molars, also frequently associated with affected permanent incisors (Weerheijm et al. 2001). Today, a more complete picture of MIH includes affected permanent canines as well (Schmalfuss et al. 2016). The definition of HSPM (idiopathic hypomineralisation of 1–4 second primary molars), was later introduced and has so far not been studied as widely as MIH (Elfrink et al. 2008).

MIH and HSPM may have a negative impact on children’s wellbeing, especially when PEB is involved. Previous studies on MIH have reported problems such as high sensitivity of the affected teeth (Fagrell et al. 2008); and reduced effect of local anaesthesia and many dental treatment sessions due to material restoration problems, which in turn may lead to the development of dental fear (Jälevik and Klingberg 2002). Higher susceptibility to dental caries in those with MIH and HSPM has also been found (Weerheijm et al. 2015).

Reported prevalence rates of MIH and HSPM vary. Among young age groups in Nordic countries, MIH has been found to range from 18% in Sweden (8-year-olds) (Jalevik et al. 2001) and 19% in Finland (7–13-year-olds) (Leppaniemi et al. 2001), to 37% in Denmark (6–8-year-olds) (Wogelius et al. 2008). In Norway, only one prevalence study to date has targeted MIH in individuals from the general population, and a prevalence of 14% was reported among the participating 16-year-olds (Schmalfuss et al. 2016). In addition, a case–control study from Norway, aiming to examine birth asphyxia and the occurrence of MIH at the age of 8–10 years, reported an MIH prevalence of 24% among cases and 25% among the controls (Sidaly et al. 2016). Regarding the prevalence of HSPM, a review of the literature published by Elfrink et al. (2015) found that the prevalence ranged between 0 and 22% worldwide. The MIH prevalence in the same report was found to range from 3 to 44%.

The aetiology of MIH and HSPM remains largely unresolved, despite an increase in studies over the last decades (Elfrink et al. 2015). In addition, the aetiology is considered to be multifactorial (Weerheijm et al. 2015). Recent systematic literature reviews focusing on MIH have suggested several risk factors, such as maternal illness and psychological stress during pregnancy; perinatal factors such as hypoxia, delivery complications (i.e., caesarean section) and prematurity; postnatal factors such as early childhood illness/fever and use of antibiotics; as well as overall exposure to environmental toxins and/or epigenetic/genetic factors (Alaluusua 2010; Silva et al. 2016; Fatturi et al. 2019; Garot et al. 2022). However, further prospective studies are still needed for the evidence on any of these factors to be conclusive.

Vitamin D plays an important role in the intestinal absorption of calcium and phosphate, which is important for the healthy development of teeth and bones (Kovacs 2021). Despite this, only a few studies have investigated the potential associations between maternal vitamin D status during pregnancy and MIH or HSPM in their offspring (van der Tas et al. 2018; Norrisgaard et al. 2019). Therefore, our study aimed to further investigate this, with the hypothesis that children of mothers with the lowest serum vitamin D levels in pregnancy would be those most affected by MIH and HSPM.

## Materials and methods

### Study design, setting and participants

The present study had a longitudinal mother and child pairs design, where data were initially collected from mothers participating in the TRaining In Pregnancy (TRIP) study, and later from a dental health examination of their children at the age of 7–9 years. Briefly, the TRIP-study was a randomised controlled trial (RCT) with 855 Norwegian women recruited from Trondheim and Stavanger University Hospitals. Its primary aim was to assess the effect of a pregnancy exercise programme in preventing gestational diabetes (Stafne et al. 2012). The participating women were recruited when attending a routine second trimester ultrasound between April 2007 and June 2009. Eligibility criteria were age ≥ 18 years and carrying a live singleton foetus. Those who had a high-risk pregnancy and/or had other diseases that could make participation problematic were excluded. Baseline maternal data were collected between gestational weeks 18–22 and follow-up data were collected between gestational weeks 32–36. Subsequently, a 7-year follow-up of the TRIP-study was conducted between 2014 and 2016. All the mothers from the TRIP-study (except 14 with an unknown address) were contacted, and those who responded also received an invitation for their child to participate in an oral health sub-study (TRIP-tann), sent in October 2015 for the children born in 2007–08 and in October 2016 for the children born in 2009.

### Assessment of maternal vitamin D status

In the original TRIP-study, fasting blood samples were taken from the mothers at the two measure points during pregnancy (gestational weeks 18–22 and 32–36). Serum analyses included measurement of 25-hydroxyvitamin-D (25(OH)D) and parathyroid hormone (PTH) by electrochemiluminescence immunoassay (ECLIA), calcium by colorimetric method, and phosphate by photometric method. All the serum analyses were conducted at Trondheim University Hospital with assays from Roche Diagnostics Ltd., Switzerland (further details have been published in Gustafsson et al. (2018)).

The serum 25(OH)D measurements at the two gestational measure points were used for the maternal vitamin D exposure variables. Serum 25(OH)D, which is a pre-hormone for the active form 1,25(OH)2D, is recognised as the best parameter to evaluate vitamin D status and reflects both vitamin D intake and ultraviolet light (UV)-exposure (Hollis 1996). Based on current nutritional guidelines, we categorised serum 25(OH)D levels < 30 nmol/l as deficient, between 30 nmol/l and 50 nmol/l as insufficient, and ≥ 50 nmol/l as sufficient (Nordic Council of Ministers 2014; Institute of Medicine 2011; Holick et al. 2011). For the data analyses, the deficient and insufficient categories were merged, resulting in a dichotomous exposure variable denoted into insufficient (< 50 nmol/l) and sufficient (≥ 50 nmol/l) maternal vitamin D levels.

Additionally, maternal nutritional intakes at the two gestational measure points were assessed through an extensive food frequency questionnaire (FFQ) (NORKOST 1997), which has previously been validated against the Norwegian population (Andersen et al. 1996, 1999, 2005). From this questionnaire, the average daily nutrient intake from food and supplements, including vitamin D and calcium, was calculated for the two gestational measure points (Elvebakk et al. 2018) and included in the current study as descriptive variables.

### Dental examination

The scoring of MIH and HSPM in the participating children was conducted by two experienced dentists in the period between May 2016 and August 2017. The two dentists were blinded to the vitamin D status of the mothers. Before the study period, both training and calibration sessions were performed based on the MIH-index, a validated assessment tool using a combination of the EAPD and mDDE indices, which has been recommended as the most appropriate for use in epidemiological studies at present (Ghanim et al. 2015, 2019; Lygidakis et al. 2022). Further details of the calibration procedure and inter- and intra-examiner results are presented in Supplementary file S1. Following the appropriate guidelines, MIH and HSPM registrations were not made if less than 1/3 of the tooth was visible, or if the hypomineralisation defects were smaller than 1 mm or located on approximal surfaces. The severity of the defects was graded according to colour (white/cream to yellow/brown), and whether PEB, atypical caries, or atypical fillings were present. Diffuse opacities were recorded, but not included in the MIH and HSPM definitions or variables. For the variable defined as MIH, both prevalence (having at least one first permanent molar with enamel hypomineralisation) and mean number of affected MIH teeth (first permanent molars and permanent incisors where at least one first permanent molar was also affected) were calculated. Similarly, for HSPM, prevalence and mean number of affected second primary molars were calculated.

### Covariates

Covariates comprised of maternal and child socio-demographic variables, maternal nutritional status during pregnancy, and maternal serum measurements (Stafne et al. 2012). Self-reported information on maternal characteristics, such as age (continuously in years), educational level (high school ≤ 13 years, university ≤ 4 years or university > 4 years), marital status (married/living with a partner or single), parity (0 or ≥ 1), smoking status (current smoker yes or no), and height and weight before current pregnancy was acquired from the TRIP-study records. Maternal body mass index (BMI) before current pregnancy was calculated as weight (kg) divided by the squared value of height (m^2^) and included as a continuous variable. Information on offspring sex (male or female) and date of birth was also acquired from the TRIP-study records. Child age at the dental exam was calculated from the date of birth and the date of the examination (continuously in years). The birth season was categorised using date of birth and based on the following groups, which have been used previously in the TRIP-study; winter (December–February), spring (March–May), summer (June–August), and autumn (September–November) (Gustafsson et al. 2018).

### Statistical analyses

Descriptive statistics and negative binomial hurdle models were used to analyse the associations between maternal vitamin D in gestational weeks 18–22 and 32–36, and MIH and HSPM in the children. Stata/MP version 15.1 for Windows (StataCorp LLC, TX, USA) was used for the descriptive analysis, reported as mean and standard deviation (SD) for continuous variables and frequency (percentage) for categorical variables. R version 4.0.2 (R Core Team 2020) and the R package ‘pscl’ were used for the regression/hurdle model analyses (Zeileis et al. 2008). Hurdle models have been recommended for analysis of dental health data, which frequently have a positively skewed distribution and may peak on zero with the large number of zero counts that are generated for non-affected participants (e.g., participants with no teeth affected by HSPM or MIH) (Hofstetter et al. 2016). Hurdle models provide both an odds ratio (OR) using logistic regression, to assess the prevalence of the outcome according to the exposure (referred to as the zero part), and a rate ratio (RR) using a negative binomial distribution, to assess the extent of the outcome among those affected (i.e., mean number of affected teeth among those with MIH or HSPM) according to the exposure (referred to as the count part). The sufficient maternal 25(OH)D exposure category was chosen as the reference group, as ≥ 50 nmol/l is recommended as sufficient serum vitamin D for the general population in the Nordic Nutrition Recommendations 2012 (Nordic Council of Ministers 2014). P values and 95% confidence intervals (95% CIs) were included to assess the statistical significance of the results, with a *p* value < 0.05 (two-sided) considered statistically significant. Potential confounders adjusted for in the multivariate analyses were chosen based on prior knowledge of relevant demographic and health-related risk factors that could potentially influence both the exposure and outcome. The factors included in the analyses were: mother’s age, education, parity, BMI before current pregnancy, season of birth for the current pregnancy, and child sex and age at the dental exam. Participants with missing values on any of the included variables were excluded from the relevant analyses.

### Ethics approval

Ethical approval from the Norwegian Regional Committees for Medical and Health Research (REK) was acquired before the initiation of the current study (2015/639/REK sør-øst), the original TRIP-study (4.2007.81), and the 7-year follow-up (2014/618/REK midt). Parents of the children participating in the dental examination gave written consent.

## Results

**Fig. 1:**
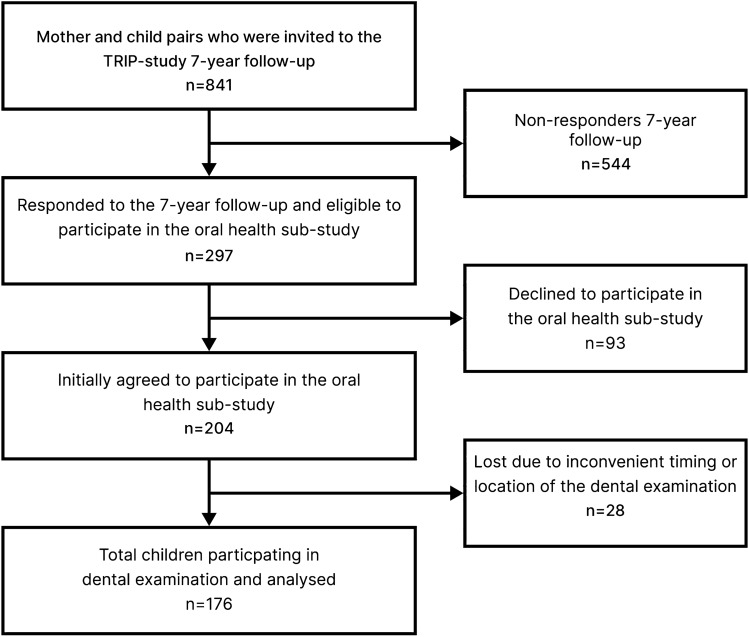
Flowchart of the study population

Among the 841 mothers available from the TRIP-study, 297 (35%) responded to the 7-year follow-up, and of those, 204 (24%) initially accepted the invitation for their child to participate in the oral health sub-study (Fig. 1). After completion of the data collection, a total of 176 children with an average age of 8.1 years (48% girls) had completed the dental examination and were included in the data analyses. The mothers of the participating children were found to be homogenous according to several background factors (such as marital status, education, and smoking status), and had similar background characteristics to the non-participating mothers from the TRIP-study (see Table 1 and Supplementary file S2). All responding and non-responding mothers were of Norwegian origin.

**Table 1:** Maternal and child characteristics by maternal serum 25(OH)D levels

	Total participants (*n* = 176)	Maternal 25(OH)D gestational week 18–22		Maternal 25(OH)D gestational week 32–36^c^	
		Insufficient < 50 nmol/l (*n* = 48)	Sufficient ≥ 50 nmol/l (*n* = 128)	Insufficient < 50 nmol/l (*n* = 52)	Sufficient ≥ 50 nmol/l (*n* = 115)
Maternal characteristics at inclusion (gestational week 18–22)					
Age, mean (SD)	31.2 (4.0)	31.2 (4.3)	31.2 (3.9)	31.3 (4.3)	31.0 (3.8)
Educational level, *n* (%)					
High school ≤ 13 years	11 (6.3)	4 (8.3)	7 (5.5)	4 (7.7)	7 (6.1)
University ≤ 4 years	71 (40.3)	17 (35.4)	54 (42.2)	15 (28.9)	52 (45.2)
University > 4 years	94 (53.4)	27 (56.3)	67 (52.3)	33 (63.5)	56 (48.7)
Marital status, *n* (%)					
Married/living with partner	174 (98.9)	48 (100.0)	126 (98.4)	52 (100.0)	113 (98.3)
Single	2 (1.1)	0 (0.0)	2 (1.6)	0 (0.0)	2 (1.7)
Parity, *n* (%)					
0	105 (59.7)	23 (47.9)	82 (64.1)	25 (48.1)	77 (67.0)
≥ 1	71 (40.3)	25 (52.1)	46 (35.9)	27 (51.9)	38 (33.0)
Smoking, *n* (%)					
No	173 (98.3)	47 (97.9)	126 (98.4)	52 (100.0)	112 (97.4)
Yes	3 (1.7)	1 (2.1)	2 (1.6)	0 (0.0)	3 (2.6)
BMI before current pregnancy, mean (SD)^a^	22.9 (3.1)	24.0 (3.5)	22.5 (2.8)	23.1 (3.7)	22.9 (2.8)
Maternal nutrient intake and serum measures at gestational week 18–22, mean (SD)					
Daily total vitamin D intake (µg)	10.6 (7.1)	8.8 (6.4)	11.3 (7.3)	–	–
Daily total calcium intake (mg)	953.9 (353.8)	891.7 (325.7)	977.3 (362.3)	–	–
Serum 25(OH)D (nmol/l)	68.4 (27.6)	37.7 (8.8)	79.9 (23.0)	–	–
Serum calcium (mmol/l)	2.27 (0.07)	2.26 (0.07)	2.27 (0.06)	–	–
Serum phosphate (mmol/l)	1.18 (0.13)	1.20 (0.15)	1.18 (0.13)	–	–
Serum PTH (pmol/l)	2.87 (1.22)	3.53 (1.70)	2.62 (0.87)	–	–
Maternal nutrient intake and serum measures at gestational week 32–36, mean (SD)					
Daily total vitamin D intake (µg)^b^	11.0 (8.0)	-	-	9.3 (7.8)	11.9 (8.1)
Daily total calcium intake (mg)^b^	951.9 (353.7)	-	-	921.5 (311.8)	960.3 (375.7)
Serum 25(OH)D (nmol/l)^c^	66.8 (29.6)	–	–	36.8 (9.4)	80.4 (25.3)
Serum calcium (mmol/l)^c^	2.25 (0.07)	–	–	2.24 (0.07)	2.25 (0.07)
Serum phosphate (mmol/l)^c^	1.17 (0.14)	–	–	1.14 (0.15)	1.19 (0.14)
Serum PTH (pmol/l)^c^	3.70 (1.73)	–	–	4.26 (1.91)	3.45 (1.59)
Child characteristics					
Sex, *n* (%)					
Male	91 (51.7)	24 (50.0)	67 (52.3)	23 (44.2)	64 (55.7)
Female	85 (48.3)	24 (50.0)	61 (47.7)	29 (55.8)	51 (44.3)
Age at dental exam, mean (SD)	8.1 (0.4)	8.0 (0.4)	8.1 (0.4)	8.1 (0.4)	8.1 (0.4)
Season of birth, *n* (%)					
Winter (Dec–Feb)	37 (21.0)	4 (8.3)	33 (25.8)	13 (25.0)	23 (20.0)
Spring (Mar–May)	44 (25.0)	15 (31.3)	29 (22.7)	20 (38.5)	20 (17.4)
Summer (Jun–-Aug)	54 (30.7)	16 (33.3)	38 (29.7)	10 (19.2)	41 (35.7)
Autumn (Sep–Nov)	41 (23.3)	13 (27.1)	28 (21.9)	9 (17.3)	31 (27.0)

^a^1 participant had missing data on BMI before current pregnancy

^b^4 participants had missing data on vitamin D and calcium intake at third trimester

^c^9 participants did not provide a blood sample at the second gestational measure point

The mean maternal serum 25(OH)D levels were 68.4 nmol/l (SD 27.6; minimum 13, maximum 152) in gestational weeks 18–22 and 66.8 nmol/l (SD 29.6; minimum 12, maximum 163) in gestational weeks 32–36 (Table 1). Nine and 13 mothers had deficient levels of 25(OH)D (< 30 nmol/l) in gestational weeks 18–22 and 32–36, respectively. After including deficient levels in the insufficient vitamin D category for data analysis purposes, approximately one-third of the participating mothers were classified into the insufficient group (< 50 nmol/l) at both of the gestational measure points (see Table 1). In addition, nine mothers did not provide a blood sample at gestational week 32–36, reducing the total sample to 167 mothers at this measure point.

As can be seen in Table 2, there were 55 children (32%) who had MIH and 39 children (22%) had HSPM. Across the total sample, a mean number of 0.91 (SD 1.74) and 0.45 (SD 0.98) teeth were affected by MIH or HSPM, respectively. Regarding severity, 31 (18%) had a yellow/brown opacity and 8 (5%) had PEB on at least one MIH or HSPM tooth. In addition, there was one child who had atypical caries on a first permanent molar, and two children had a first permanent molar with an atypical filling.

**Table 2:** Distribution of MIH and HSPM by maternal serum 25(OH)D levels

	Total participants (*n* = 176)	Maternal 25(OH)D gestational week 18–22		Maternal 25(OH)D gestational week 32–36^a^	
		Insufficient < 50 nmol/l (*n* = 48)	Sufficient ≥ 50 nmol/l (*n* = 128)	Insufficient < 50 nmol/l (*n* = 52)	Sufficient ≥ 50 nmol/l (*n* = 115)
MIH^b^					
Present (> 0), *n* (%)	55 (31.8)	12 (25.0)	43 (34.4)	13 (25.0)	37 (32.7)
Mean (SD)	0.91 (1.74)	0.96 (2.14)	0.89 (1.57)	0.81 (2.0)	0.88 (1.59)
HSPM					
Present (> 0), *n* (%)	39 (22.2)	13 (27.1)	26 (20.3)	14 (26.9)	23 (20.0)
Mean (SD)	0.45 (0.98)	0.54 (0.99)	0.41 (0.98)	0.46 (0.87)	0.45 (1.05)
Yellow/brown opacity on MIH^b^ or HSPM tooth					
Present (> 0), *n* (%)	31 (17.6)	8 (16.7)	23 (18.0)	8 (15.4)	21 (18.3)
Mean (SD)	0.32 (0.81)	0.29 (0.77)	0.33 (0.82)	0.29 (0.85)	0.32 (0.79)
PEB on MIH^b^ or HSPM tooth					
Present (> 0), *n* (%)	8 (4.6)	1 (2.1)	7 (5.5)	3 (5.8)	5 (4.4)
Mean (SD)	0.05 (0.21)	0.02 (0.14)	0.06 (0.23)	0.06 (0.24)	0.04 (0.21)

The mean (SD) values include those with and without teeth affected by MIH or HSPM

^a^9 participants did not have a 25(OH)D measurement from the second gestational measure point

^b^3 participants had no erupted first permanent molars. Permanent incisors with hypomineralisation were included if the participant also had an affected first permanent molar

In the adjusted count part of the hurdle model, a significantly higher number of affected teeth were found among those with MIH in the insufficient maternal vitamin D group measured between gestational weeks 18–22 than those with MIH in the sufficient maternal vitamin D group (Table 3). There were no significant differences found between the insufficient and sufficient maternal vitamin D groups at either of the two gestational measure points and number of affected teeth among those with HSPM. Furthermore, no statistically significant associations concerning maternal vitamin D measured in gestational week 18–22 or 32–36 and prevalence of having at least one tooth with MIH or HSPM (the zero part of the hurdle model) were found.

**Table 3:** Regression analyses with maternal 25(OH)D levels and MIH and HSPM in the offspring at 7–9 years of age

	Disease prevalence (zero part)				Disease extent (count part)			
	Unadjusted OR (95% CI)	*p* value	Adjusted OR^c^ (95% CI)	*p* value	Unadjusted RR (95% CI)	*p* value	Adjusted RR^c^ (95% CI)	*p* value
Maternal 25(OH)D gestational week 18–22								
MIH^b^								
Insufficient (< 50 nmol/l)	0.64 (0.30–1.35)	*p* = 0.24	0.56 (0.24–1.29)	*p* = 0.17	1.68 (0.99–2.86)	*p* = 0.06	1.82 (1.13–2.93)	*p* = 0.01
Sufficient (≥ 50 nmol/l)	Ref		Ref		Ref		Ref	
HSPM								
Insufficient (< 50 nmol/l)	1.46 (0.68–3.14)	*p* = 0.34	1.30 (0.55–3.05)	*p* = 0.55	0.97 (0.53–1.78)	*p* = 0.92	0.94 (0.47–1.88)	*p* = 0.85
Sufficient (≥ 50 nmol/l)	Ref		Ref		Ref		Ref	
Maternal 25(OH)D gestational week 32–36^a^								
MIH^b^								
Insufficient (< 50 nmol/l)	0.69 (0.33–1.44)	*p* = 0.32	0.48 (0.20–1.13)	*p* = 0.09	1.31 (0.70–2.44)	*p* = 0.40	1.41 (0.77–2.59)	*p* = 0.27
Sufficient (≥ 50 nmol/l)	Ref		Ref		Ref			
HSPM								
Insufficient (< 50 nmol/l)	1.47 (0.69–3.17)	*p* = 0.32	1.41 (0.60–3.34)	*p* = 0.43	0.62 (0.32–1.20)	*p* = 0.16	0.76 (0.35–1.65)	*p* = 0.48
Sufficient (≥ 50 nmol/l)	Ref		Ref		Ref		Ref	

^a^9 participants did not have a 25(OH)D measurement from the second gestational measure point

^b^3 participants had no erupted first permanent molars, and were therefore not included in the regression analysis for the MIH outcome. Two participants had two erupted first permanent molars, and one participant had one erupted first permanent molar, and these participants were still included in the regression analysis. Permanent incisors with hypomineralisation were included if the participant also had an affected first permanent molar

^c^Adjusted for mother’s age, education and parity at inclusion, BMI before current pregnancy, season of birth current pregnancy, and child sex and age. One participant was excluded because of missing values on BMI before current pregnancy

## Discussion

In this longitudinal mother–child pairs study, we investigated potential associations between maternal vitamin D levels during pregnancy and offspring MIH and HSPM at 7–9 years of age. Consistent with our hypothesis, we found that among those with MIH, the children with insufficient maternal vitamin D levels in gestational weeks 18–22 had a higher number of affected teeth, than those with sufficient maternal vitamin D. Furthermore, we found no significant differences in the prevalence of having at least one tooth affected by MIH or HSPM and maternal vitamin D at either of the two gestational measure points (the zero part of the hurdle model).

The prevalence of both MIH and HSPM found in this study population was reasonably high, when compared to previous studies (Jalevik et al. 2001; Leppaniemi et al. 2001; Elfrink et al. 2015). Potential explanations for this could be first, that we investigated MIH within the recommended period (i.e., most were 8 years of age); second, that we included demarcated opacities down to 1 mm in the HSPM and MIH variables; and third, that we used examiners experienced in paediatric dentistry, and they received extensive training and calibration in recording HSPM and MIH according to recommended guidelines (Weerheijm et al. 2003; Lygidakis et al. 2022).

As far as we are aware, this is the first study to investigate potential associations between maternal serum vitamin D during pregnancy and number of affected teeth among those with MIH or HSPM (the count part of the hurdle model). For the logistic (zero) part of our analyses, there is one previous longitudinal study from the Netherlands that is comparable. Consistent with our results, this previous study did not find any significant associations between maternal serum 25(OH)D levels at mid-gestation, or in cord-blood, and prevalence of having at least one tooth with MIH or HSPM among 6-year-old children (van der Tas et al. 2018).

Tooth development starts prenatally, with the second primary molars initiating enamel mineralisation around gestational week 18, and the crowns are normally completed within the first year of life. The first permanent molars initiate mineralisation around the time of birth, and therefore overlap with the mineralisation of the second primary molars for some time (Koch et al. 2017). As tooth mineralisation initiates during pregnancy, it is plausible that maternal vitamin D levels may affect enamel mineralisation in the offspring. This is supported by previous studies that have found associations between maternal prenatal serum 25(OH)D levels and other oral health outcomes, such as enamel hypoplasia and dental caries in the primary dentition (Schroth et al. 2014; Reed et al. 2019).

In our study, we found that insufficient maternal vitamin D levels were associated with the number of affected teeth among those with MIH. This may indicate that unfavourable maternal vitamin D levels in pregnancy could also create a disadvantageous basis for permanent tooth development, even though the first permanent teeth do not initiate mineralisation before around the time of birth. Previous studies have indicated that infants may be more reliant on vitamin D to maintain adequate calcium levels after birth than during foetal life, where there are additional mechanisms in place to help maintain adequate calcium levels (Kovacs 2021). Furthermore, the vitamin D level in the foetus has been shown to correlate with the mother’s, and therefore, if the mother has insufficient levels during pregnancy, the levels in the newborn will also be inadequate (Hollis and Pittard 1984). Additionally, the sources of vitamin D are limited after birth and often reliant on being provided with adequate supplementation, especially if breastfed, making those with already low vitamin D stores from birth particularly vulnerable (Kovacs 2021).

A recent Danish study, which conducted a post hoc analysis of data from a randomised controlled trial, found that children of mothers receiving a daily high-dose vitamin D supplement (2400 IU vitamin D_3_) from gestational week 24 to 1 week postpartum, had a significantly reduced risk of enamel hypomineralisation in both second primary molars and permanent molars at 6 years of age (Norrisgaard et al. 2019). This supports the argument that adequate maternal vitamin D during pregnancy could be an advantage for both primary and permanent tooth development. However, due to the limited number of published studies on this topic, further studies are needed to investigate whether our finding is replicable and to clarify the role of maternal vitamin D during pregnancy in regard to child tooth development.

Strengths of the study included measurement of serum 25(OH)D at two different time points during tooth development, that two meticulously calibrated dentists performed the examinations, and that most of the children were examined at 8 years of age (the recommended age for MIH examination) (Weerheijm et al. 2003). Also, we used hurdle modelling, which accommodates data with a large number of zero counts, and provides the ability to investigate potential differences in the number of MIH and HSPM teeth among those affected in the exposure groups, as well as potential differences in prevalence. A further strength of the study was the availability of prospectively collected data, including the exposure, outcomes, and potential confounders, reducing the possibility of recall bias and reverse causality. In addition, as the participating mothers were homogenous with respect to several potentially confounding factors, the study qualifies for a reasonably good internal validity. However, as with any observational study, there is still a possibility of unmeasured and residual confounding.

The main limitation of the study was the low response rate, which may have limited our power to find significant differences between the exposure groups in relation to some of the outcome variables. This may partly explain why we did not find any significant differences regarding the association between maternal vitamin D and HSPM, of which there were a lower number of cases and affected teeth than MIH. In addition, there were 9 mothers with missing blood samples at the second gestational measure point, which further reduced the sample size, and may have increased the possibility of selection bias in this part of the analysis. Notably, a considerable proportion of the mothers with a missing blood sample at the second gestational measure point had children with MIH (*n* = 5 in total), resulting in 10 MIH teeth not being included in that part of the analysis.

Regarding the external generalisability of our results, the sample of our study was found to have similar characteristics to the original TRIP-study sample (see Supplementary file S2). However, although the original TRIP-study sample itself was found to be reasonably representative of the general pregnant population in Norway, there were some differences that were also reflected in our study sample (e.g., they had lower BMIs, were more physically active, and were less ethnically diverse) (Stafne et al. 2012). Nonetheless, limited external generalisability could be argued to mainly pose a problem in surveys or studies where the primary aim is to report prevalence estimates. While in studies where the main goal is to study potential causal associations and achieve scientific generalisability, a homogenous sample may provide better control for confounding factors (Rothman 2014).

## Conclusion

Considering any limitations of the present study, it has been shown that insufficient maternal serum vitamin D at mid-pregnancy was associated with a higher number of affected teeth among the offspring with MIH at 7–9 years of age. Further, large prospective studies are needed to investigate whether this finding is replicable and to clarify the potential role of maternal serum vitamin D levels during pregnancy and MIH, as well as HSPM, in children.

## Supplementary Information

Below is the link to the electronic supplementary material.

Supplementary file1 (DOCX 22 KB)

Supplementary file2 (DOCX 27 KB)

## Data Availability

The datasets generated and analyzed during the current study are not publicly available due to the Norwegian Health Research Act not allowing public data sharing unless the research participants have explicitly given their consent to do so. In the current study, the participants did not consent to this. However, the data are available from the corresponding author on reasonable request.
