# Supplementary material for: Maternal vitamin D status in pregnancy and molar incisor hypomineralisation and hypomineralised second primary molars in the offspring at 7–9 years of age: a longitudinal study
Source: Eur Arch Paediatr Dent. 2022 May 12;23(4):557–66. doi: 10.1007/s40368-022-00712-y (PMC9338139; doi:10.1007/s40368-022-00712-y)
Supplement: Supplementary file 1 — Supplementary file1 (DOCX 22 KB) [file 40368_2022_712_MOESM1_ESM.docx]

Maternal vitamin D status in pregnancy and Molar-Incisor Hypomineralisation and Hypomineralised Second Primary Molars in the offspring at 7-9 years of age: a longitudinal study

European Archives of Paediatric Dentistry

Torunn Børsting^1,2^, Annemarie Schuller^3,4^, Paula van Dommelen^3^, Signe Nilssen Stafne^2,10^, Marit S. Skeie^5,1^, Anne B. Skaare^6^, Siv Mørkved^2,10^, Kjell Å. Salvesen^7,11^, Astrid Kamilla Stunes^7,12^, Mats Peder Mosti^7,12^, Miriam K. Gustafsson^9,7^, Unni Syversen^7,8^, Tone Natland Fagerhaug^1,2^

1) Center for Oral Health Services and Research, Mid-Norway (TkMidt), Trondheim, Norway

2) Department of Public Health and Nursing, Norwegian University of Science and Technology (NTNU), Trondheim, Norway

3) Department of Child Health, the Netherlands Organization for Applied Scientific Research (TNO), Leiden, The Netherlands

4) Centre of Dentistry and Oral Hygiene, University Medical Center Groningen, University of Groningen, Groningen, The Netherlands

5) Department of Clinical Dentistry, University of Bergen, Bergen, Norway

6) Department of Paediatric Dentistry and Behavioural Science, Faculty of Dentistry, University of Oslo, Oslo, Norway

7) Department of Clinical and Molecular Medicine, Norwegian University of Science and Technology (NTNU), Trondheim, Norway

8) Department of Endocrinology, Trondheim University Hospital (St. Olavs Hospital), Trondheim, Norway

9) Regional Education Center (RegUt), Helse Midt-Norge, Trondheim, Norway

10) Department of Clinical Service, Trondheim University Hospital (St. Olavs Hospital), Trondheim, Norway

11) Department of Obstetrics and Gynaecology, Trondheim University Hospital (St Olavs Hospital), Trondheim, Norway

12) Medical Clinic, Trondheim University Hospital (St Olavs Hospital), Trondheim, Norway

Correspondence: Torunn Børsting, [torbo@tkmidt.no](mailto:torbo@tkmidt.no)

**Supplimentary file S1.** Calibration procedure and results

Calibration procedure

Prior to the dental examination period, the two experienced examiners underwent calibration courses for scoring MIH and HSPM, including theory and training. The gold standard was set by consensus of two specialists in paediatric dentistry (ABS and MSS). Test 1, 2 and 3 measured inter-examiner reliability. Test 1 was based on 84 photos of tooth surfaces with various types of enamel defects and healthy surfaces. Test 2, conducted at a different time point, was based on 70 photos out of the 84 used in Test 1. In both these two sessions, the two examiners’ results were compared with the gold standard. The average scores of the two examiners against the gold standard are provided in the results for Test 1 and 2 below. In the result for Test 3, the inter-examiner results between the two examiners at the two calibration time points were calculated. Finally, intra-examiner reliability was assessed in Test 4, where each examiner’s own assessments of same photos (n=70) at the two time-points were compared.

Calibration results

The following mean unweighted Cohen’s kappa results were achieved:

- Test 1: 0.73 against the gold standard for the first calibration.
- Test 2: 0.71 against the gold standard for the second calibration.
- Test 3: 0.58 and 0.60 for the first and second calibration, respectively.
- Test 4: 0.60 and 0.59 for the first and second examiner, respectively.
