# Supplementary material for: Maternal vitamin D status in pregnancy and molar incisor hypomineralisation and hypomineralised second primary molars in the offspring at 7–9 years of age: a longitudinal study
Source: Eur Arch Paediatr Dent. 2022 May 12;23(4):557–66. doi: 10.1007/s40368-022-00712-y (PMC9338139; doi:10.1007/s40368-022-00712-y)
Supplement: Supplementary file 2 — Supplementary file2 (DOCX 27 KB) [file 40368_2022_712_MOESM2_ESM.docx]

Maternal vitamin D status in pregnancy and Molar-Incisor Hypomineralisation and Hypomineralised Second Primary Molars in the offspring at 7-9 years of age: a longitudinal study

European Archives of Paediatric Dentistry

Torunn Børsting^1,2^, Annemarie Schuller^3,4^, Paula van Dommelen^3^, Signe Nilssen Stafne^2,10^, Marit S. Skeie^5,1^, Anne B. Skaare^6^, Siv Mørkved^2,10^, Kjell Å. Salvesen^7,11^, Astrid Kamilla Stunes^7,12^, Mats Peder Mosti^7,12^, Miriam K. Gustafsson^9,7^, Unni Syversen^7,8^, Tone Natland Fagerhaug^1,2^

1) Center for Oral Health Services and Research, Mid-Norway (TkMidt), Trondheim, Norway

2) Department of Public Health and Nursing, Norwegian University of Science and Technology (NTNU), Trondheim, Norway

3) Department of Child Health, the Netherlands Organization for Applied Scientific Research (TNO), Leiden, The Netherlands

4) Centre of Dentistry and Oral Hygiene, University Medical Center Groningen, University of Groningen, Groningen, The Netherlands

5) Department of Clinical Dentistry, University of Bergen, Bergen, Norway

6) Department of Paediatric Dentistry and Behavioural Science, Faculty of Dentistry, University of Oslo, Oslo, Norway

7) Department of Clinical and Molecular Medicine, Norwegian University of Science and Technology (NTNU), Trondheim, Norway

8) Department of Endocrinology, Trondheim University Hospital (St. Olavs Hospital), Trondheim, Norway

9) Regional Education Center (RegUt), Helse Midt-Norge, Trondheim, Norway

10) Department of Clinical Service, Trondheim University Hospital (St. Olavs Hospital), Trondheim, Norway

11) Department of Obstetrics and Gynaecology, Trondheim University Hospital (St Olavs Hospital), Trondheim, Norway

12) Medical Clinic, Trondheim University Hospital (St Olavs Hospital), Trondheim, Norway

Correspondence: Torunn Børsting, [torbo@tkmidt.no](mailto:torbo@tkmidt.no)

**Supplementary file S2.** Comparison of background variables for participants in the current study and non-respondents from original TRIP-study

|  | **Total participants current study (n=176)** | **Non-respondents from original study (n=679)** |
| --- | --- | --- |
| **Maternal characteristics at inclusion (gestational week 18-22)** |  |  |
| Age, mean (SD) | 31.2 (4.0) | 30.3 (4.4) |
| Education, n (%) |  |  |
| High school <=13 years | 11 (6.3) | 84 (12.4) |
| University <=4 years | 71 (40.3) | 260 (38.3) |
| University >4 years | 94 (53.4) | 335 (49.3) |
| Marital status, n (%) |  |  |
| Married/living with partner | 174 (98.9) | 660 (97.4) |
| Single | 2 (1.1) | 18 (2.6) |
| Parity, n (%) |  |  |
| 0 | 105 (59.7) | 383 (56.4) |
| ≥1 | 71 (40.3) | 296 (43.6) |
| Smoking, n (%) |  |  |
| No | 173 (98.3) | 673 (99.1) |
| Yes | 3 (1.7) | 6 (0.9) |
| Weight before current pregnancy (kg), mean (SD)^a^ | 64.8 (9.5) | 66.1 (9.9) |
| **Maternal nutrient intake and serum measures at gestational week 18-22^b^** |  |  |
| Daily total vitamin D intake (µg), mean (SD) | 10.6 (7.1) | 10.4 (7.0) |
| Daily total calcium intake (mg), mean (SD) | 953.9 (353.8) | 978.5 (375.7) |
| Serum 25(OH)D (nmol/l), mean (SD) | 68.4 (27.6) | 65.5 (24.0) |
| Serum 25(OH)D (nmol/l) catergorised, n (%) |  |  |
| Deficient (<30 nmol/l) | 9 (5.1) | 31 (4.6) |
| Insufficient (≥30 & <50 nmol/l) | 39 (22.2) | 153 (22.6) |
| Sufficient (≥50 & <75 nmol/l) | 62 (35.2) | 261 (38.5) |
| Optimal (≥75 nmol/l) | 66 (37.5) | 233 (34.4) |
| Serum Calcium (mmol/l), mean (SD) | 2.27 (0.07) | 2.27 (0.07) |
| Serum Phosphate (mmol/l), mean (SD) | 1.18 (0.13) | 1.20 (0.12) |
| Serum PTH (pmol/l), mean (SD) | 2.87 (1.22) | 2.75 (1.1) |
| **Maternal nutrient intake and serum measures at gestational week 32-36^c^** |  |  |
| Daily total vitamin D intake (µg), mean (SD) | 11.0 (8.0) | 10.1 (7.1) |
| Daily total calcium intake (mg), mean (SD) | 951.9 (353.7) | 963.7 (338.4) |
| Serum 25(OH)D (nmol/l), mean (SD) | 66.8 (29.6) | 63.5 (26.2) |
| Serum 25(OH)D (nmol/l) catergorised, n (%) |  |  |
| Deficient (<30 nmol/l) | 13 (7.8) | 39 (7.0) |
| Insufficient (≥30 & <50 nmol/l) | 39 (23.4) | 155 (27.8) |
| Sufficient (≥50 & <75 nmol/l) | 58 (34.7) | 192 (34.5) |
| Optimal (≥75 nmol/l) | 57 (34.1) | 171 (30.7) |
| Serum Calcium (mmol/l), mean (SD) | 2.25 (0.07) | 2.25 (0.07) |
| Serum Phosphate (mmol/l), mean (SD) | 1.17 (0.14) | 1.17 (0.14) |
| Serum PTH (pmol/l), mean (SD) | 3.70 (1.73) | 3.58 (1.4) |
| ^a^ 7 missing among non-respondents from original sample | | |
| ^b^ 1 missing for serum analyses at gestational week 18-22 among non-respondents from original sample | | |
| ^c^ Nutrient intake at gestational week 32-36 lost to follow-up: 4 in current study sample, 90 among non-respondents from original sample | | |
| ^c^ Serum analyses at gestational week 32-36 missing or lost to follow-up: 9 in current study sample, 122 among non-respondents from original sample | | |
